# Modulation of Skin Inflammatory Responses by Aluminum Adjuvant

**DOI:** 10.3390/pharmaceutics15020576

**Published:** 2023-02-08

**Authors:** Yanhang Liao, Lixiang Sun, Meifeng Nie, Jiacheng Li, Xiaofen Huang, Shujun Heng, Wenlu Zhang, Tian Xia, Zhuolin Guo, Qinjian Zhao, Ling-juan Zhang

**Affiliations:** 1 State Key Laboratory of Cellular Stress Biology, School of Pharmaceutical Sciences, Xiamen University, Xiamen 361002, China; 2 State Key Laboratory of Molecular Vaccinology and Molecular Diagnostics, National Institute of Diagnostics and Vaccine Development in Infectious Diseases, School of Public Health, Xiamen University, Xiamen 361002, China; 3 Department of Dermatology, Shanghai Tenth People’s Hospital, Tongji University School of Medicine, Shanghai 200443, China; 4 College of Pharmacy, Chongqing Medical University, Chongqing 400016, China

**Keywords:** vaccine adjuvant, aluminum salt, psoriasis, atopic dermatitis, inflammation, T cell polarization

## Abstract

Aluminum salt (AS), one of the most commonly used vaccine adjuvants, has immuno-modulatory activity, but how the administration of AS alone may impact the activation of the skin immune system under inflammatory conditions has not been investigated. Here, we studied the therapeutic effect of AS injection on two distinct skin inflammatory mouse models: an imiquimod (IMQ)-induced psoriasis-like model and an MC903 (calcipotriol)—induced atopic dermatitis-like model. We found that injection of a high dose of AS not only suppressed the IMQ-mediated development of T-helper 1 (Th1) and T-helper 17 (Th17) immune responses but also inhibited the IMQ-mediated recruitment and/or activation of neutrophils and macrophages. In contrast, AS injection enhanced MC903-mediated development of the T-helper 2 (Th2) immune response and neutrophil recruitment. Using an in vitro approach, we found that AS treatment inhibited Th1 but promoted Th2 polarization of primary lymphocytes, and inhibited activation of peritoneal macrophages but not bone marrow derived neutrophils. Together, our results suggest that the injection of a high dose of AS may inhibit Th1 and Th17 immune response-driven skin inflammation but promote type 2 immune response-driven skin inflammation. These results may provide a better understanding of how vaccination with an aluminum adjuvant alters the skin immune response to external insults.

## 1. Introduction

The skin, the primary interface with the environment, functions as an important barrier, protecting the body against pathogens, chemicals, allergens, and mechanical insults. Prolonged exposure of epidermal keratinocytes to environmental insults may lead to activation of the immune system, and ultimately the development of inflammatory skin disorders such as psoriasis and atopic dermatitis (AD), the most common dermatologic conditions [1,2].

T cells, the central component of adaptive immunity, play a critical role in host defense against pathogens. During infections, the cytokine milieu modulates the differentiation and polarization of CD4^+^ T cells into distinct effector phenotypes, including interferon γ (IFNγ) producing-T-helper 1 (Th1) cells that mediate the clearance of infected cells, interleukin 4 (IL4)- and IL13-producing Th2 cells that play a role in parasite expulsion and driving allergic response, and IL17-producing Th17 cells that mediate anti-fungal response and promote autoimmunity [3,4]. Psoriasis and atopic dermatitis are considered to be T cell-mediated chronic relapsing inflammatory skin diseases, mediated by different effector mechanisms. Psoriatic inflammation is primarily driven by the Th17 immune response, although Th1 cells are also present [5]. In contrast, the overactivation of Th2 cells plays a dominant role in driving acute skin inflammation in atopic dermatitis [1,5,6,7,8].

In the early 1900s, investigators found that injecting aluminum-precipitated antigen induced a stronger antibody and protective immune response compared to the response generated by free antigen injection [9,10]. Aluminum salts have now become one of the most commonly used adjuvants in human vaccines. Despite their longstanding use, the mechanisms by which aluminum adjuvants enhance immune responses are still not fully understood. Studies have shown that aluminum adjuvants promote the differentiation of CD4 T cells into Th2 effector cells but do not support Th1 cell differentiation in vivo or in vitro [11,12,13,14].

Dysregulation of T cell differentiation is the central disease mechanism for both psoriasis and atopic dermatitis, while aluminum salts exhibit their potential immunomodulatory effect through effector T cell differentiation/activation. Therefore, we aimed to determine whether the administration of aluminum salt influences the activation of the immune system in mouse models of psoriasis or atopic dermatitis. We also tested the in vitro effect of aluminum salt on effector T cell differentiation and the activation of key myeloid cells (neutrophils and macrophages). The results of our study provide insights into how vaccination may lead to the development of skin side effects by altering the skin’s immune response to external insults.

## 2. Materials and Methods

### 2.1. Chemicals, Antibodies and Dyes

Aluminum salt, a suspension of aluminum hydroxyphosphate containing 1680 μg aluminum/mL and 9.33 mM/L inorganic phosphorus, is prepared by mixing AlCl3, Na2HPO4, and NaOH (to adjust the PH to 6~7) as described previously [15,16,17]. The HiScript II Q RT SuperMix kit for RNA reverse-transcription was purchased from Vazyme (Nanjing, China); 2× SYBR Green qPCR Master Mix for quantitative reverse transcription PCR was purchased from Bimake (Houston, TX, USA). Imiquimod Cream (IMQ) was purchased from the Med-Shine Corporation (Hongkong). Methotrexate disodium (MTX) and MC903 (calcipotriol) were purchased from Selleckchem (Houston, TX, USA). Rat anti-Ki67 antibody and ProLong™ Gold Antifade Mountant with DAPI (4′,6-diamidino-2-phenylindole, dihydrochloride) were purchased from ThermoFisher Scientific (Waltham, MA, USA). Alexa Fluor 647 AffiniPure Donkey Anti-Rat IgG (H+L) secondary antibody was purchased from Jackson ImmunoResearch (West Grove, PA, USA). Zombie violet viability dye, PECy7 anti-CD45, APC anti-CD11C, PerCP-Cy5.5 anti-LY6C, PECy7 anti-CD11B, APC/Cyanine7 anti-CD3, AF488 anti-IL4 and APC anti-TCRγ/δ antibodies were purchased from BioLegend (San Diego, CA, USA); CD16/CD32 Monoclonal Antibody, AF488 anti-LY6G, PE anti-F4/80, AF700 anti-MHCII, AF488 anti-IL13, PerCP-Cy5.5 anti-IFNγ, AF700 anti-CD4 antibodies and the fixation and permeabilization buffer set for fluorescence activated cell sorting (FACS) analysis were purchased from ThermoFisher Scientific. A hematoxylin and eosin staining kit was obtained from ZSGB-BIO Corporation (Beijing, China). Recombinant mouse IL-4 was obtained from BioLegend. Recombinant mouse IL-2, mouse IL-12, mouse IFNγ, mouse IL-6, mouse IL1β, mouse TGFβ1 were purchased from R&D Systems (Minneapolis, MN, USA). LPS and FSL were purchased from ThermoFisher Scientific.

### 2.2. Animal Cares and Animal Models

C57BL/6 mice used in this study were purchased from GemPharmatech (Nanjing, China), then bred and maintained in the standard pathogen-free (SPF) environment of the Laboratory Animal Center in Xiamen University. All animal experiments were approved by the Institutional Animal Care and Use Committee of Xiamen University. For the IMQ-induced psoriasis-like model, 7~8-week-old female C57BL/6 mice were anesthetized, shaved, and depilated. Then, the backs of the mice were treated daily with a topical dose of 50 mg IMQ cream for 7 days. A single dose of aluminum salt (500 μL volume containing 840 μg AL^3+^) was injected peritoneally at day 2.5 post IMQ application, and daily i.p. injection of methotrexate (MTX), a commonly used systemic anti-inflammatory drug to treat psoriasis, was used as a positive control [18]. For the MC903-induced dermatitis-like model, the backs of the mice were shaved, depilated, and treated daily with a topical dose of 45 μL MC903 (100 μM, dissolved in ethanol) for 10 days. A single dose of aluminum salt was injected peritoneally at day 4 post-MC903 application. Daily administration of methotrexate (MTX) at 1 mg/kg was used as a positive anti-psoriatic agent [18]. The appearances of the lesions were recorded using a digital camera.

The severity of skin inflammation was evaluated daily, including the measurements for skin redness, scaling, and epidermal thickness, and scored separately using a five-point scale from 0 to 4 (0, no incidence; 1, slight; 2, moderate; 3, marked; and 4, most severe) [19,20]. Additionally, the cumulative score (redness plus scaling plus epidermal thickness) was depicted [19,20].

### 2.3. Histology and Immunohistochemistry (IHC)

The biopsies of lesional skin were embedded in OCT (#4583, SAKURA, Torrance, CA, USA) followed by frozen sectioning. Frozen mouse skin sections were subjected to hematoxylin and eosin staining according to the manufacturer’s protocol. For IHC staining, OCT-embedded sections were permeabilized with 0.1% saponin (#47036, Sigma, Tokyo, Japan) for 10 min, then blocked in 5% BSA (#4240GR100, Biofroxx, Einhausen, Germany) solution for 1 h. Blocked sections were incubated with the indicated primary antibodies at 4 °C overnight, followed by appropriate fluorophore-coupled secondary antibodies in the dark for 4–6 h at 4 °C. Finally, the sections were mounted and observed using a Zeiss LSM 880 Laser Confocal Microscope (Zeiss, Jena, Germany).

### 2.4. Flow Cytometry and Analysis (FACS)

FACS analysis was performed to analyze immune cell populations of innate immunity and adaptive immunity in inflammatory skin. Briefly, skin tissues were digested with collagenase D (V900893, Sigma) and Dnase 1 (D8071, Solarbio, Beijing, China) to prepare a single cell suspension. The cell suspension was then stained with zombie violet viability dye, and blocked non-specific Fc-mediated interactions with CD16/CD32 Monoclonal Antibody. Then, cells were incubated with the indicated antibody cocktail mix (Panel A or B, listed in Table 1) for 1 h at 4 °C with shaken every 10 min gently. Finally, the cells were resuspended in stabilizing fixative buffer (#338036, BD biosciences, San Jose, CA, USA). FACS analysis was performed using the Thermo Attune NxT machine (Waltham, MA, USA) and further analyzed using FlowJo V10 software.

### 2.5. Quantitative Reverse Transcription-Quantitative PCR (qRT-PCR) Analyses

Total cellular RNA was extracted from skin tissues or cultured cells using Trizol (#T9424, Sigma), chloroform, and the RNAExpress Total RNA Kit (#M050, NCM Biotech, Newport, UK), and purified RNA was reversed transcribed to cDNA using the HiScript II Q RT SuperMix kit. Quantitative PCRs were performed by SYBR Green qPCR Master Mix (#B21202, Bimake) on the Qtower real-time machine (Analytikjena, Swavesey, Cambridge, UK). All primers used in our study were designed to span exon—exon junctions and are shown in Table 2. The expression of the *Tbp* (TATA-Box Binding Protein) gene was used as a housekeeping gene to normalize the target gene expression. The ratio of target mRNA to *Tbp* was calculated using the −2^ΔCT^ (ΔC_T_ = C_T_ of target gene—C_T_ of *Tbp*) method based on the published relative quantification method [21].

### 2.6. In Vitro T Cell Differentiation

Firstly, naïve T cells were isolated and purified from the inguinal lymph nodes of 8-week wild-type mice, and the cells were seeded in a 24-well cell culture plate that was pre-coated overnight with anti-CD3&CD28 (3 μg/mL). Next, T cells were incubated with the indicated cytokine cocktail mix including rm-IL-12 (15 ng/mL), rm-Il-2 (10 ng/mL) and anti–IL-4 (10 μg/mL) for Th1 differentiation, or rm-IL-4 (10 ng/mL), rm-IL-2 (10 ng/mL), anti-IFN-γ (15 μg/mL) and anti-IL-12 (15 μg/mL) for Th2 development, or rm-IL-6 (20 ng/mL), rm-TGFβ1 (2 ng/mL) and rm-IL-1β (10 ng/mL) to promote Th17 differentiation, with AS (1:250 dilution, ~6.74 μg/mL Al^3+^). The cells were cultured at 37 °C for 144 h. Then the cells were stimulated with PMA (50 ng/mL), Ionomycin (500 ng/mL), and GolgiPlug for 3 h. Finally, the cell suspensions were centrifuged, and the supernatant and precipitated cells were collected and used for further experiments.

### 2.7. Neutrophil and Macrophage Cultures

To isolate neutrophils, bone marrow cells were first flushed from mouse femurs and tibias using RPMI-1640 medium containing 10% FBS. Bone marrow cells were treated with red blood cell lysis buffer and washed once with PBS. Cells were then overlaid on top of the Histopaque gradient (1077–1119) and centrifuged for 30 min at 872× *g* at room temperature, without a break. Neutrophils were collected at the interface of the Histopaque 1119 and Histopaque 1077 layers, and isolated neutrophils were cultured in RPMI-1640 medium containing 10% FBS and 1% penicillin/streptomycin, and then treated as indicated. The purity of neutrophils was >90% as determined by flow cytometry. For treatment, cells were pretreated with AS (1:250 dilution from the 1680 μg/mL stock = 6.74 μg/mL Al^3+^) for 2 h and then stimulated with FSL (50 ng/mL) for 6 h.

Peritoneal macrophages were isolated by injecting the mice with 5 mL of PBS containing 1% FBS, gently massaging the belly, and then aspirating the fluid. This process was repeated three times in total. The cells were treated with red blood cell lysis buffer and resuspended in DMEM containing 10% FBS and 1% penicillin/streptomycin. Isolated macrophages were seeded at a cell density of 5 × 10^5^ cells per well of a 24-well plate. Twenty-four hours after being seeded, nonadherent cells were removed, and the adherent macrophages were incubated with DMEM containing 10% FBS and 1% penicillin/streptomycin for all experiments. For treatment, cells were pretreated with AS (1:250 or 1:1000 dilution from the 1680 ug AL^3+^/mL stock) for 2 h and then stimulated with LPS (0.5 μg/mL) for 12 h.

### 2.8. Statistics

**Figure 1:**
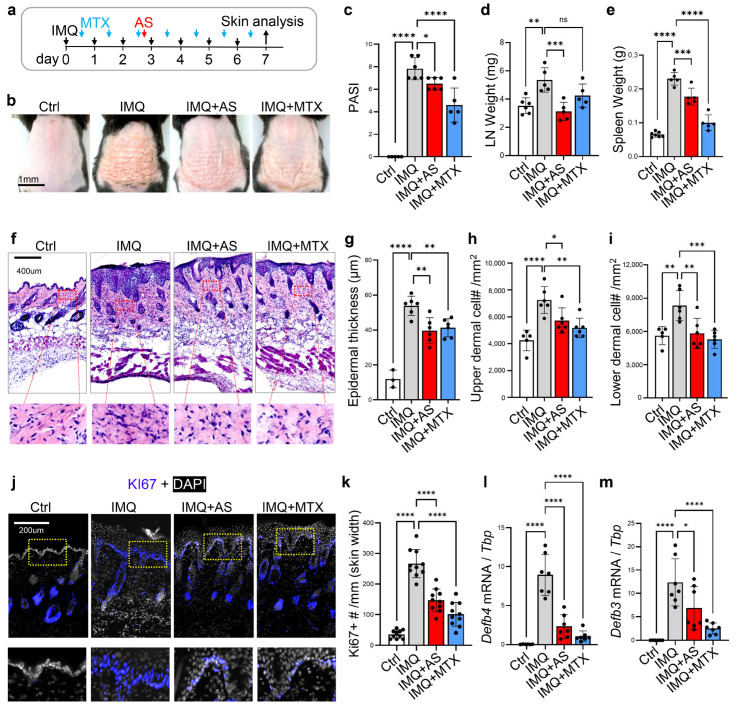
Administration of aluminum salt alleviates the IMQ-triggered development of psoriasis-like skin inflammation in mice: (**a**) Overview of the experimental setting. Psoriasis-like skin inflammation was triggered by daily topical application of IMQ to mouse dorsal skin for 7 days with or without AS injection between day 3~4 or daily MTX injection from day 0~1 as indicated; (**b**) Representative skin images for each group at day 7; (**c**) Bar graphs showing quantified Psoriasis Area and Severity Index (PASI) scores for each group (*n* = 5~6 mice/group); (**d**,**e**) The weight of lymph node (LN) or Spleen at day of sample collection (*n* = 5~6 mice/group); (**f**) HE staining of skin sections from each group; (**g**) Bar graphs showing quantified epidermal thickness for each group (*n* = 3~6 fields/group); (**h**,**i**) Bar graphs showing quantified upper dermal or lower dermal cell density for each group as indicated (*n* = 5~6/group); (**j**) Immunostaining of skin sections with anti-Ki67 (blue) and nuclei were counter stained by DAPI (white). Zoom-in images are shown in the lower panel. (**k**). Bar graphs showing quantified number of Ki67+ epidermal cells per mm width of the skin (*n* = 10 fields/group); (**l**,**m**) qRT-PCR analysis of the mRNA expression levels of *Defb4* and *Defb3* (ratios to HK gene *Tbp* were shown, *n* = 6~7/group). All error bars indicate the mean ± SEM. * *p* < 0.05, and statistical analysis was performed by one-way ANOVA analysis, followed by Dunnett test (**d**,**e**,**g**,**f**,**i**,**k**,**l**,**m**) or a two-stage linear step-up procedure of Benjamini, Krieger, and Yekutieli (**c**) for multiple comparisons. * *p* < 0.05, ** *p* < 0.01, *** *p* < 0.001, **** *p* < 0.0001, ns, no-significant.

Experiments were repeated at least 3 times independently. All statistical analyses were performed using GraphPad Prism version 9 software. For comparisons between more than two groups, statistical analysis was performed by one-way analysis of variance (ANOVA) followed by a Dunnett test [22], or two way ANOVA followed by a Bonferroni test [23], to correct for multiple comparisons as listed in the legend. For Figure 1b, a one-way ANOVA analysis was performed, followed by a two-stage linear step-up procedure of Benjamini, Krieger, and Yekutieli to correct for multiple comparisons by controlling the False Discovery Rate [24]. Quantitative results are presented as mean ± standard error of mean (SEM). A *p* value less than 0.05 was considered statistically significant and indicated with asterisks, * *p* < 0.05, ** *p* < 0.01, *** *p* < 0.001, **** *p* < 0.0001.

## 3. Results

### 3.1. Administration of Aluminum Salt Alleviates the Development of Psoriasis-like Skin Inflammation in Mice

To determine the effect of an aluminum adjuvant (see characterization results in Figure S1a–d) on the development of skin inflammation, we first employed an imiquimod (IMQ)-induced psoriasis-like skin inflammation mouse model (Figure 1a), in which both Th1 and Th17 cell-mediated adaptive immunity play a role in driving epidermal hyperplasia and scaling [25]. A dose of 840 μg aluminum/mouse was used in our animal study, and this dose is within the recommended range in vaccines for clinical use [26]. It is also a common practice for determining vaccine immunogenicity in a mouse model as an in vivo potency assay, in which a full human dose or even doubled the human dose was used for intraperitoneal (*i.p.*) injection into each mouse [27,28]. The injection of this high dose of AS had a minimal systemic cytotoxic effect in mice (Figure S1e,f). We found a single *i.p.* injection of aluminum salt (AS) during the application of IMQ was effective in alleviating the development of psoriasis phenotypes characterized by erythema, thickness, and scales (Figure 1b). However, it was less effective than the daily i.p. injection of methotrexate (MTX) (Figure 1b), a commonly used systemic anti-inflammatory drug for psoriasis. In addition, AS injections blocked the development of systemic inflammatory responses, as shown by the reduced enlargement of the lymph nodes and spleen tissues (Figure 1d,e). AS was more effective than MTX in reducing lymph node enlargement (Figure 1d).

Histological analysis of the skin sections revealed that epidermal thickness and dermal cell infiltration in IMQ-treated skin were significantly lower in AS- and MTX-treated mice (Figure 1f–i). Immunostaining for Ki67, a proliferation-associated nuclear antigen, showed that IMQ treatment increased the number of Ki67+ basal and/or supra-basal keratinocytes, and this increase in epidermal cell proliferation was partially inhibited by either AS or MTX injections (Figure 1j,k). Antimicrobial peptides, including defensins (DEFBs), are strongly induced in activated keratinocytes in psoriasis, and defensins participate in cutaneous inflammation by promoting keratinocyte migration, proliferation, and the production of inflammatory cytokines [29]. Analysis of the expression of key defensins, including *Defb3*, *Defb4,* and *Defb14*, showed that AS or MTX partially inhibited the IMQ-mediated induction of defensin genes (Figure 1l,m and Figure S1g). Interestingly, we found that the expression of type 1 collagen (Col1a1) was significantly reduced in IMQ-treated skin, and this effect was reversed by AS or MTX injections (Figure S1h), suggesting that IMQ-triggered dysregulation of dermal homeostasis can be restored by AS. Together, these results demonstrate that the administration of aluminum salt alleviates the development of psoriasis-like skin inflammation in mice.

### 3.2. Administration of Aluminum Salt Inhibited the Development of Th1 and Th17 Immune Responses in the IMQ-Induced Psoriasis Model

**Figure 2:**
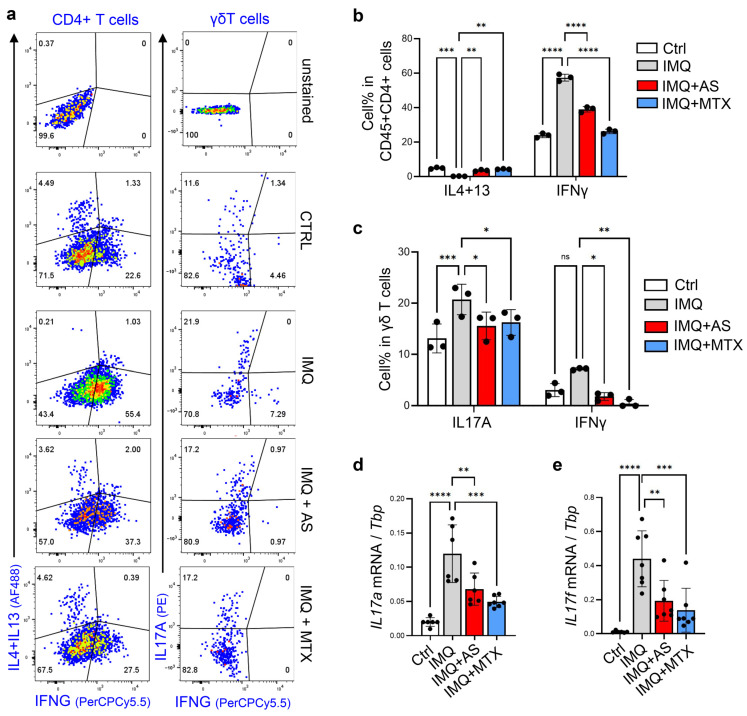
Administration of aluminum salt inhibited the development of Th1 and Th17 immune response in the IMQ-induced psoriasis model: (**a**–**c**) FACS plots (**a**) and quantified bar graphs (**b**,**c**) showing the percentage of IL4/IL13^+^, IFNγ^+^ or IL17A^+^ cells in CD45^+^ CD4^+^ T cells (**b**) or CD45^+^ γδ T cells (**c**) in the skin (*n* = 3/group); (**d**,**e**) qRT-PCR analysis of the mRNA expression levels of *Defb4* and *Defb3* (ratios to HK gene *Tbp* were shown, *n* = 6~7/group). All error bars indicate mean ± SEM, and statistical analysis was performed by two-way ANOVA with Bonferroni-corrected multiple comparisons (**b**,**c**) or one-way ANOVA analysis with Dunnett-corrected multiple comparisons (**d**,**e**). * *p* < 0.05, ** *p* < 0.01, *** *p* < 0.001, **** *p* < 0.0001, ns, non-significant.

Next, we aimed to investigate the effect of AS on lymphocyte activation in an IMQ-induced psoriasis model. In mouse skin, γδ T cells are the major IL17-producing cells after infection, wounding or IMQ application. On the other hand, αβ T cells, either CD4^+^ or CD8^+^, are capable of producing large amounts of the Th1 cytokine IFNγ or Th2 cytokines (IL4 and IL13) under different inflammatory conditions [8,30]. FACS analysis of skin CD4 T cells revealed that IMQ treatment inhibited Th2 but promoted Th1 polarization of CD4+ T cells in the skin, and increased the percentage of IL17A-producing γδ T cells (Figure 2a–c). These effects were largely reversed by either AS or MTX injections (Figure 2a–c). In line with these FACS results, qRT-PCR analysis showed that the IMQ-dependent induction of *Il17a* and *Il17f* was also significantly inhibited by either AS or MTX injections (Figure 2d,e). However, the IMQ-mediated induction of Il23 and Il22 did not appear to be influenced by AS or MTX injections (Figure S2a,b).

### 3.3. Administration of Aluminum Salt Reduced the Recruitment and Activation of Myeloid Cells

**Figure 3:**
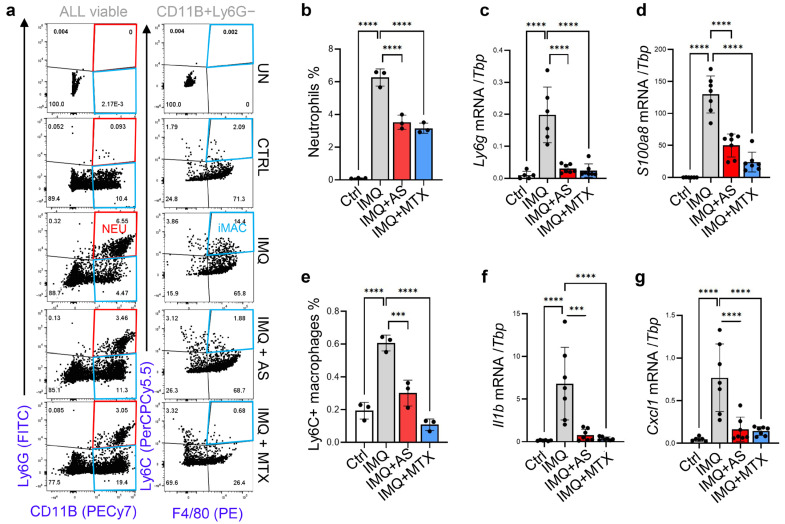
Administration of aluminum salt reduced the recruitment and activation of myeloid cells in the IMQ-induced psoriasis model: (**a**) FACS plots showing the presence of neutrophils (CD11B+Ly6G+) and inflammatory macrophages (CD11B+Ly6G-F4/80+Ly6C+); (**b**) Quantified FACS results showing the percentage of neutrophils in each group (*n* = 3/group); (**c**,**d**) qRT-PCR analysis of the mRNA expression levels of *Ly6g* and *S100a8* (ratios to HK gene *Tbp* were shown, *n* = 6~7/group); (**e**) Quantified FACS results showing the percentage of inflammatory macrophages in each group (*n* = 3/group); (**f**,**g**) qRT-PCR analysis of the mRNA expression levels of *Il1b* and *Cxcl1* (ratios to HK gene *Tbp* were shown, *n* = 6~7/group). All error bars indicate mean ± SEM, and statistical analysis was performed by one-way ANOVA analysis with Dunnett-corrected multiple comparisons. *** *p* < 0.001, **** *p* < 0.0001.

Abnormal dermal infiltration and activation of myeloid cells, including neutrophils and macrophages, is one of the histologic hallmarks of psoriasis [31]. Flow cytometry (FACS) analysis showed that AS or MTX injections reduced the percentage of infiltrated CD11B^+^Ly6G^+^ neutrophils in IMQ-treated skin samples (Figure S3a and Figure 3a,b). In line with this FACS result, qRT-PCR analysis showed that the expression levels of neutrophil marker genes, including Ly6g and S100A8, were also reduced in skin samples from AS or MTX-treated mice (Figure 3c,d). FACS analysis of CD11B+F4/80+ macrophages revealed that in IMQ-treated skin, macrophages expressed Ly6C (Figure S3a and Figure 3a,e), a phenotypic marker for pro-inflammatory macrophages [32], indicating that macrophages shift from a resting to an inflammatory state during the development of psoriasis. Furthermore, we found that the IMQ-mediated increase in Ly6C^hi^ macrophages was inhibited by either AS or MTX injection (Figure 3a,e). Additionally, IMQ-mediated induction of Il1b, Cxcl1, and Saa3, key genes related to myeloid cell activation and/or chemotaxis [33,34], was also significantly inhibited by AS or MTX injections (Figure 3a,e and Figure S3b). Together these results show that the systemic administration of aluminum salt potently suppresses the activation of myeloid cells, including neutrophils and macrophages, in the IMQ-induced psoriasis model.

### 3.4. Administration of Aluminum Salt Promoted the Development of MC903-Induced Atopic Dermatitis-like Skin Inflammation

**Figure 4:**
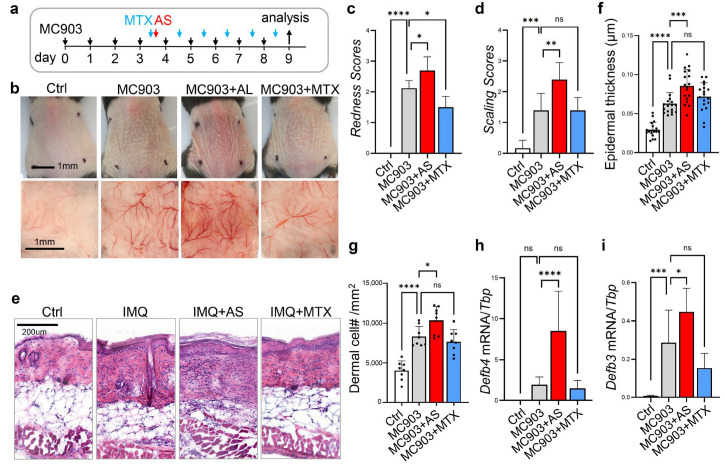
Administration of aluminum salt promoted the development of MC903-induced atopic dermatitis-like skin inflammation: (**a**) Overview of the experimental setting. Atopic dermatitis-like skin inflammation was triggered by daily topical application of MC903 to mouse dorsal skin for 9 days with or without AS injection between day 3~4 or daily MTX injection from day 3~4 as indicated; (**b**) Representative skin images for each group at day 9; (**c**) Bar graphs showing quantified redness scores for each group (*n* = 5 mice/group); (**d**) Bar graphs showing quantified scaling scores for each group; (**e**) HE staining of skin sections from each group (*n* = 5 mice /group); (**f**) Bar graphs showing quantified epidermal thickness for each group (*n* = 15~20 fields/group); (**g**) Bar graphs showing quantified dermal cell density for each group (*n* = 8 fields/group); (**h**,**i**) qRT-PCR analysis of the mRNA expression levels of *Defb4* and *Defb3* (ratios to housekeeping gene *Tbp* were shown, *n* = 6~8/group). All error bars indicate mean ± SEM, and statistical analysis was performed by one way ANOVA analysis with Dunnett-corrected multiple comparisons. * *p* < 0.05, ** *p* < 0.01, *** *p* < 0.001, **** *p* < 0.0001, ns, non-significant.

To determine whether aluminum salt promotes the development of the Th2 immune response in a mouse model of dermatitis, we adapted the MC903-induced dermatitis model, one of the most well-characterized murine models of atopic dermatitis in which T cells are preferentially polarized toward the Th2 phenotype [35]. Daily topical application of MC903 to the back skin led to reddening and scaling of the skin, which could be partially inhibited by systemic administration of MTX; in contrast, MC903-induced redness and scaling were markedly increased by the injection of aluminum salt (Figure 4a–d). Histological analysis of skin sections revealed that epidermal thickness and dermal cell infiltration in MC903-treated skin were significantly higher in the AS injected mice (Figure 4e–g). In addition, qRT-PCR analysis showed that AS injection enhanced the MC903-mediated induction of genes associated with epidermal keratinocyte activation (*Defb4* and *Defb3*) (Figure 4h,i). Similar to the IMQ-induced psoriasis model, MC903 application also led to suppression of *Col1a1* expression in the skin, but AS injection failed to restore *Col1a1* expression in MC903-treated skin (Figure S4a).

### 3.5. Effect of Aluminum Salt Injection in Modulating the MC903-Mediated Activation of T Cells or Myeloid Cells

#### 3.5.1. Administration of Aluminum Salt Promoted the Development of Type 2 Immune Response in the MC903-Induced Dermatitis Model

**Figure 5:**
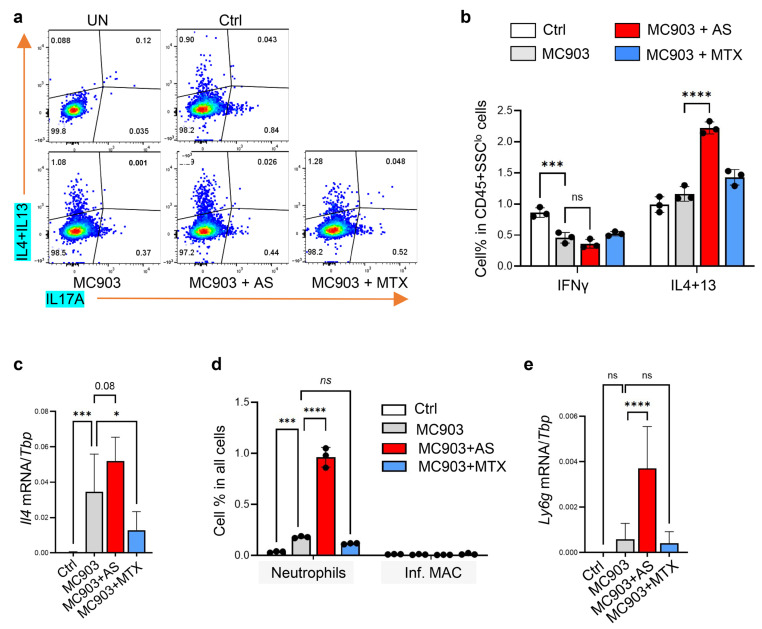
Effect of aluminum salt injection in modulating the MC903-mediated activation of T cells or myeloid cells: (**a**,**b**) FACS plots (A) and quantified bar graphs (**b**) showing the percentage of IFNγ+ or IL4/IL13+ cells in CD45^+^SSC^lo^ T cells (*n* = 3/group); (**c**) qRT-PCR analysis of the mRNA expression level of *Il4* (ratios to HK gene *Tbp* were shown, *n* = 6~8/group); (**d**) Bar graphs showing the percentage of neutrophils (CD11B+Ly6G+) and inflammatory macrophages (inf. MAC; F4/80+Ly6C+) in all cells (quantified from FACS plots shown in Figure S5A, *n* = 3/group). (**e**) qRT-PCR analysis of the mRNA expression level of Ly6g (*n* = 6~8/group). All error bars indicate mean ± SEM, and statistical analysis was performed by two-way ANOVA with Bonferroni-corrected multiple comparisons (**b**,**d**) or one-way ANOVA analysis with Dunnett-corrected multiple comparisons (**c**,**e**). * *p* < 0.05, *** *p* < 0.001, **** *p* < 0.0001, ns, non-significant.

FACS analysis of skin cells revealed that AS injection significantly enhanced the expression of Th2 cytokines (IL4 and IL13) from CD45^+^SSC^lo^ T cells in MC903-treated skin (Figure 5a,b). In addition, qRT-PCR analysis showed that MC903-mediated induction of Il4 was further enhanced by AS injection but inhibited by MTX injection (Figure 5c). These results showed that the administration of aluminum salt promoted the development of type 2 inflammation in the MC903-induced dermatitis mouse model.

#### 3.5.2. Administration of Aluminum Salt Altered Myeloid Cell Activation in the MC903-Induced Dermatitis Model

FACS analysis of myeloid cells showed that, in contrast to daily IMQ application, daily application of MC903 led to only a small increase in CD11B^+^Ly6G^+^ neutrophils and no increase in CD11B^+^F4/80^+^Ly6C^+^ pro-inflammatory macrophages (Figure S5a and Figure 5d). AS injection increased MC903-mediated infiltration of neutrophils, but not the inflammatory macrophages (Figure S5a and Figure 5d). In line with the FACS results, qRT-PCR analysis showed that AS injection increased the MC903-mediated induction of *Ly6g* (neutrophil marker gene) (Figure 5e). These results showed that the administration of aluminum salt promoted the infiltration of neutrophils, but not inflammatory macrophages, into MC903-treated skin.

### 3.6. The In Vitro Effect of Aluminum Salt in Modulating T Cell Differentiation and Myeloid Cell Activation

We showed that injection of AS promoted the development of type 2 skin inflammation, and differentially altered myeloid cell activation in two distinct dermatitis models in vivo. Next, we aimed to determine whether AS directly alters the activation of lymphocytes and myeloid cells in vitro.

#### 3.6.1. Aluminum Salt Inhibited Th1 but Promoted Th2 Polarization during In Vitro T Cell Differentiation

**Figure 6:**
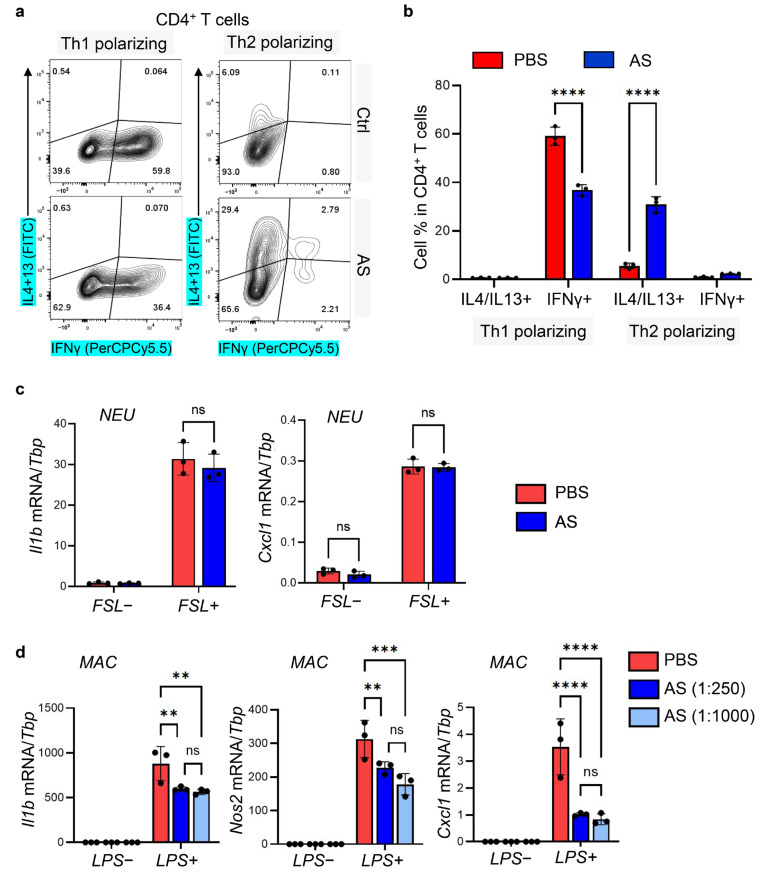
The in vitro effect of aluminum salt in modulating T cell differentiation and myeloid cell activation: (**a**) FACS plots analyzing the expression of IFNγ and IL4/IL13 in primary CD4+ T cells cultured under Th1- or Th2- polarizing conditions with or without AS (1:250). (**b**) quantified bar graphs showing the percentage of IL4/IL13+ or IFNγ+ CD4+ T cells (*n* = 3/group); (**c**) Primary neutrophils isolated from bone marrow were stimulated with TLR2 agonist FSL with PBS or AS (1:250), and cells were subjected to qRT-PCR analysis of *Il1b* or *Cxcl1* mRNA expression as indicated (*n* = 3/group); (**d**) Primary peritoneal macrophages were stimulated with TLR4 agonist LPS with PBS or AS (1:250 or 1:1000 as indicated), and cells were subjected to qRT-PCR analysis of *Il1b*, *Cxcl1,* or *Nos2* mRNA expression as indicated (*n* = 3/group). All error bars indicate mean ± SEM, and statistical analysis was performed by two-way ANOVA with Bonferroni-corrected multiple comparisons. ** *p* < 0.01, *** *p* < 0.001, **** *p* < 0.0001, ns, non-significant.

First, to determine whether aluminum salt can directly alter Th1 or Th2 polarization in differentiating lymphocytes, we subjected lymph node-derived primary lymphocytes to in vitro differentiation assays in the presence of CD3/CD28 antibody under Th1, Th2, or Th17 polarizing conditions. We found that the addition of AS significantly inhibited IFNγ production under Th1 polarizing conditions from CD4+ T cells, but promoted IL4 and IL13 production under Th2 polarizing conditions from CD4+ T cells (Figure 6a,b and Figure S6a,b). In addition, we found that under Th2 polarizing conditions, AS treatment promoted a Th17 to Th2 shift in γδ T cells, the major IL17-producing cell type in the skin (Figure S6c,d). However, under Th17 skewing conditions, in which γδ T cells were robustly shifted into IL17A producing cells, AS treatment only mildly reduced the expression of IL17A in γδ T cells without altering the expression of IFNγ or IL4/IL13 (Figure S6e,f).

#### 3.6.2. Aluminum Salt Inhibited Macrophage but Not Neutrophil Activation In Vitro

Next, primary neutrophils derived from bone marrow were stimulated with FSL, and primary peritoneal macrophages were stimulated with LPS with or without AS (Figure 6c,d). We found that while AS had no effect on neutrophil activation (Figure 6c), it significantly suppressed macrophage activation even at low concentration (1:1000 dilution from the original stock), as shown by qRT-PCR analysis of *Il1b*, *Cxcl1* and *Nos2* (Figure 6d). IL1β and NOS2 are well-established markers for inflammatory M1 macrophages [36], indicating that AS may directly inhibit macrophage polarization toward the pro-inflammatory state.

## 4. Discussion

Injection of vaccines, composed of immunogens, preservatives, adjuvants, and by-products, can elicit adverse skin reactions, such as localized or generalized eczema vaccinia, in susceptible individuals [37,38]. Conflicting results have been reported regarding the relationship between vaccination and the development of atopic diseases [39,40,41], but none of these studies investigated the specific immunomodulatory effects of the individual component of the vaccines.

Aluminum salts are widely used as adjuvants in preventive vaccines, enhancing their immunogenicity and effectiveness by stimulating a type 2 immune response [11,12]. Theoretically, AS could increase the risk of type 2 lymphocyte-mediated allergic and hyper-responsive diseases, such as atopic dermatitis. On the other hand, AS application may have a therapeutic effect against psoriasis, dominated by Th1 and Th17 cells, which are antagonistic to Th2 cells. To rest this theory, in the present study, we investigated the immuno-modulatory effect of aluminum salt in two distinct murine models of inflammatory skin diseases. We found that the injection of aluminum salt into the IMQ-induced psoriasis mouse model promoted T cell polarization from the Th1/Th17 to Th2 phenotype, suppressed neutrophil recruitment and macrophage activation, and therefore suppressed the development of psoriasis-like skin inflammation. In contrast, injection of aluminum salt promoted the development of the Th2 immune response and clinical phenotype of dermatitis in the MC903-induced atopic dermatitis-like mouse model.

Our results indicate that AS application may inhibit the Th1/Th17-mediated activation of autoimmunity but enhance the Th2-mediated activation of the allergic immune response in the skin. In line with our results, it has been reported that patients receiving subcutaneous allergen-specific immunotherapy with aluminum adjuvants are associated with a lower risk of autoimmune diseases, including psoriasis [42]. In contrast, several reports have shown that although rare, patients can develop delayed hypersensitivity or allergic cutaneous reactions to vaccines containing aluminum salts [43,44,45,46,47].

By in vitro primary culture, we showed that the addition of aluminum salt promoted Th2 differentiation and inhibited the Th1 differentiation of naïve lymphocytes. To our knowledge, this is the first study investigating the direct effect of aluminum salt on naive T cell differentiation/polarization. Furthermore, we found that the addition of aluminum salt inhibited macrophage polarization toward the pro-inflammatory state but had no direct effect on neutrophil activation. AS-mediated differential effects on neutrophil recruitment in the IMQ- or MC903-induced dermatitis models were likely indirectly mediated by AS-dependent changes in T cell effector immune responses.

It has been shown that aluminum hydroxide has a high adsorption capacity for endotoxins and LPS (283 μg/mg of Al), whereas endotoxins are electrostatically repelled by aluminum phosphate [48]. As a result, the adsorption capacities of phosphate-treated aluminum hydroxide or aluminum phosphate are only 23 and 3 μg endotoxin/mg of Al, respectively [48]. Here we report that the AS solution (which is a mixture of aluminum hydroxide and aluminum phosphate) can inhibit the inflammatory activity of LPS (0.5 μg/mL) even at low concentration (~1.7 μg AL^3+^/mL), which should only absorb 5~29 ng/mL LPS in theory. Therefore, AS-mediated absorption/neutralization of LPS may contribute to the inhibitory effect of AS against LPS, but it is unlikely the major mechanism for this inhibition. Future studies are still needed to determine the mechanism underlying the anti-inflammatory effect of AS against the LPS-mediated inflammatory response in macrophages.

A limitation of our study is that we administered aluminum salt as a single intraperitoneal injection instead of multiple intramuscular injections as performed in routine vaccinations in clinics. In addition, we investigated the immunomodulatory effect of only one high dose of AS in mouse dermatitis models, although this dose was chosen based on our and others’ previous studies in the mouse potency assay of human vaccines with aluminum adjuvants [15,16,17,27,28]. Our study offers a clue for the immunomodulatory role of aluminum, not an effective therapy for human disease. Future studies are needed to determine the optimal injection method and dose to observe the immunomodulatory function of AS in animal dermatitis models.

Together, our results provide new mechanisms underlying the immunomodulatory effect of aluminum salt on immune cell activation. Systemic injection of a high dose of aluminum adjuvant may inhibit or promote skin inflammation, depending on the involvement of specific effector T cells and/or myeloid cells during disease pathogenesis.

## Figures and Tables

**Table 1:** Antibodies used for flow cytometry and analysis.

Panel A: Immune Cell Analysis	Panel B: T Cell Analysis
APC anti-CD11C	AF488 anti-IL4 *
PerCP-Cy5.5 anti-LY6C	APC anti-TCRγ/δ
PECy7 anti-CD11B	PerCP-Cy5.5 anti-IFNγ *
APC/Cyanine7 anti-CD3	AF700 anti-CD4
AF488 anti-LY6G	AF488 anti-IL13 *
PE an-ti-F4/80	PECy7 anti-CD45
AF700 anti-MHCII	APC/Cyanine7 anti-CD3

* IL4, IL13 and IFNγ are intracellular antigens.

**Table 2:** List of primers used for qRT-PCR analyses.

Gene	Strand	Primer Sequence
*Tbp*	Forward	CCTTGTACCCTTCACCAATGAC
	Reverse	ACAGCCAAGATTCACGGTAGA
*Defb4*	Forward	TGGTGCTGCTGTCTCCACTTGC
	Reverse	AGGGCACGGACCCCAGCATA
*Defb3*	Forward	GGTGCTGCTGTCTCCACCTGC
	Reverse	TGCACCGATTCCAGCATCTGCC
*Il17a*	Forward	ACGCGCAAACATGAGTCCAGGG
	Reverse	TGAGGGATGATCGCTGCTGCCT
*Il17f*	Forward	ACGTGAATTCCAGAACCGCT
	Reverse	TGATGCAGCCTGAGTGTCTG
*Ly6g*	Forward	GACTTCCTGCAACACAACTACC
	Reverse	ACAGCATTACCAGTGATCTCAGT
*S100a8*	Forward	AAATCACCATGCCCTCTACAAG
	Reverse	CCCACTTTTATCACCATCGCAA
*Il1b*	Forward	GAAATGCCACCTTTTGACAGTG
	Reverse	TGGATGCTCTCATCAGGACAG
*Cxcl1*	Forward	CACCCGCTCGCTTCTCTG
	Reverse	TCTTGAGGTGAATCCCAGCC
*Il4*	Forward	GAGCCATATCCACGGATGCGAC
	Reverse	ATGCGAAGCACCTTGGAAGCCC
*Nos2*	Forward	GTTCTCAGCCCAACAATACAAGA
	Reverse	GTGGACGGGTCGATGTCAC
*Col1a1*	Forward	GCTCCTCTTAGGGGCCACT
	Reverse	ATTGGGGACCCTTAGGCCAT
*Il22*	Forward	CCTACATGCAGGAGGTGGTG
	Reverse	AAACAGCAGGTCCAGTTCCC
*Il23*	Forward	ATGCTGGATTGCAGAGCAGTA
	Reverse	ACGGGGCACATTATTTTTAGTCT
*Saa3*	Forward	TGCCATCATTCTTTGCATCTTGA
	Reverse	CCGTGAACTTCTGAACAGCCT
*Defb14*	Forward	TGGTGCCTGCTCCAGGGGAC
	Reverse	CAGCACACCGGCCACCTCTT

## Data Availability

Not applicable.

## Supplementary Materials

The following supporting information can be downloaded at: https://www.mdpi.com/article/10.3390/pharmaceutics15020576/s1, Figure S1: Characterization of the aluminum salt solution, and investigation of the therapeutic effect of AS on the development of psoriasis-like skin inflammation in mice (Supplemental for main Figure 1); Figure S2: The effect of aluminum salt injection in modulating T cell activation in the IMQ-induced psoriasis model (Supplemental for main Figure 2); Figure S3: Administration of aluminum salt reduced the recruitment and activation of myeloid cells in the IMQ-induced psoriasis model (Supplemental for main Figure 3); Figure S4: The effect of aluminum salt injection in modulating the development of MC903-induced atopic dermatitis-like skin inflammation (Supplemental for main Figure 4); Figure S5: The effect of aluminum salt injection in modulating the MC903-mediated activation of T cells or myeloid cells; Figure S6: The in vitro effect of aluminum salt in modulating T cell differentiation and myeloid cell activation.
