# Supplementary material for: Modulation of Skin Inflammatory Responses by Aluminum Adjuvant
Source: Pharmaceutics. 2023 Feb 8;15(2):576. doi: 10.3390/pharmaceutics15020576 (PMC9966661; doi:10.3390/pharmaceutics15020576)
Supplement: Supplementary file 1 [file pharmaceutics-15-00576-s001.zip › pharmaceutics-2178278-supplementary.pdf]

pharmaceutics

MDPI

Supplementary Materials: Modulation of Skin Inflammatory Responses by Aluminum Adjuvant

Yanhang Liao, Lixiang Sun, Meifeng Nie, Jiacheng Li, Xiaofen Huang, Shujun Heng, Wenlu Zhang, Tian Xia, Zhuolin Guo, Qinjian Zhao and Ling-juan Zhang

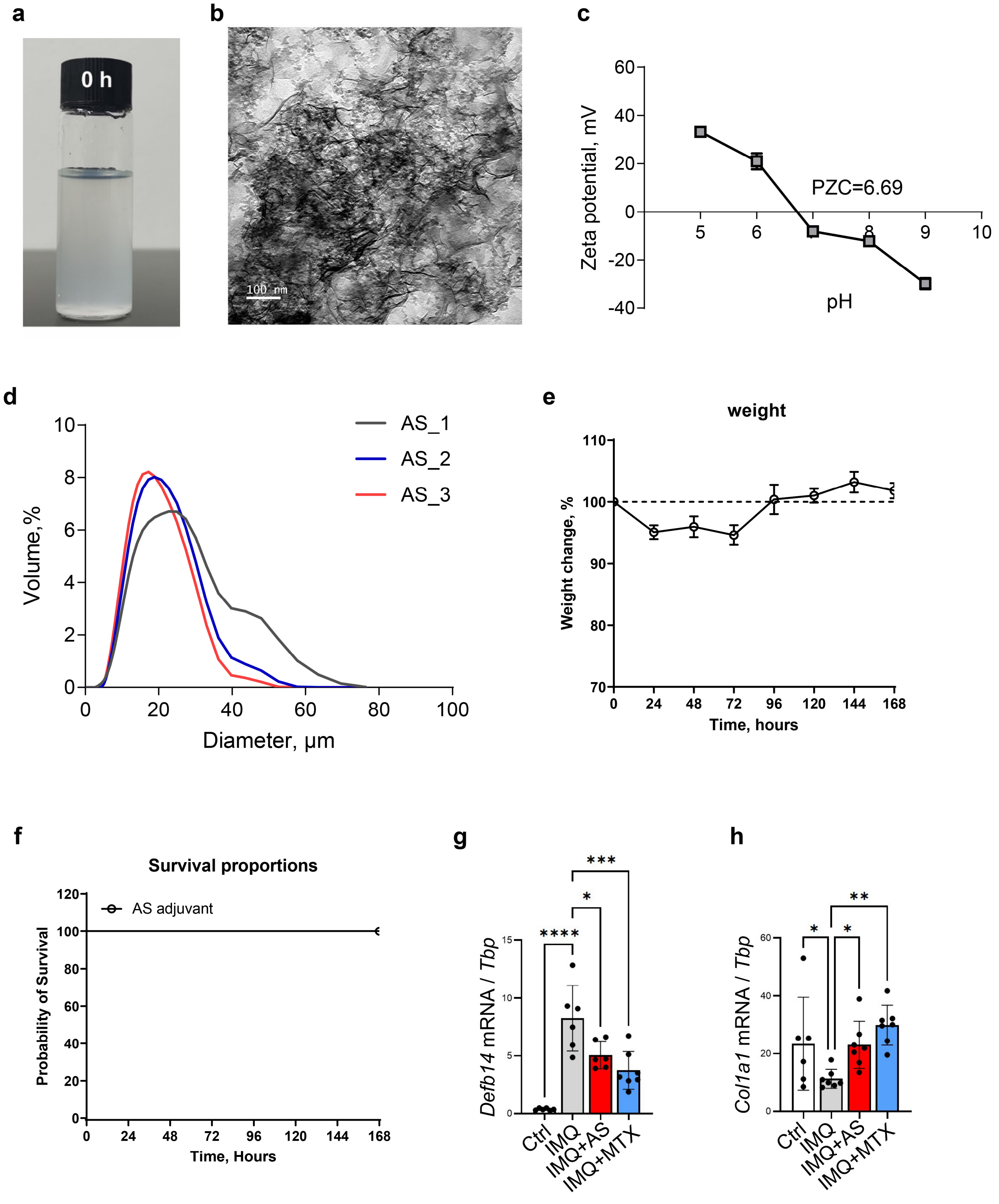

Figure S1. Characterization of the aluminum salt solution, and investigation of the therapeutic effect of AS on the development of psoriasis-like skin inflammation in mice (Supplemental for main Figure 1). (**a**) Photograph showing how aluminum salts form precipitates in solution. (**b**) Transmission electron microscopic showing that aluminum salts form filamentous structure in solution. (**c**) Zeta potentials of aluminum salts were measured at different pH using NanoBrook Omni zeta potential analyzer (Brookhaven). The potential of zero charge (PZC) values was 6.69 as shown. At physiological conditions (pH=7), AS solution carried a weak negative charge. (**d**) Measurement of particle size distribution of AS by a laser diffraction particle size analyzer (LS13320, Beckman-Coulter). Each measurement was repeated three times and the average mean particle size was recorded. As shown in the graph, the average particle size of AS was about 20 um. (**e-f**) Safety assessment of the AS adjuvant. Mice (n = 5 per group, 18~22 g) were injected intraperitoneally with 0.5 ml of the AS adjuvant (each mice received 840 ug $AL3^+$). Body weight (e) and survival rate (f) and were monitored for 7 days. After 7 days, all mice recover full weight within 7 days and no mice were found dead during experiment. (**g-h**) qRT-PCR analysis of the mRNA expression levels of *Defb14* or *Col1a1* (ratios to HK gene *Tbp* were shown) as indicated. All error bars indicate mean ± SEM (n=6~7/group) , and statistical analysis was performed by one-way ANOVA analysis with Dunnett-corrected multiple comparisons. *p < 0.05, **p < 0.01, ***p < 0.001, ****p < 0.0001.

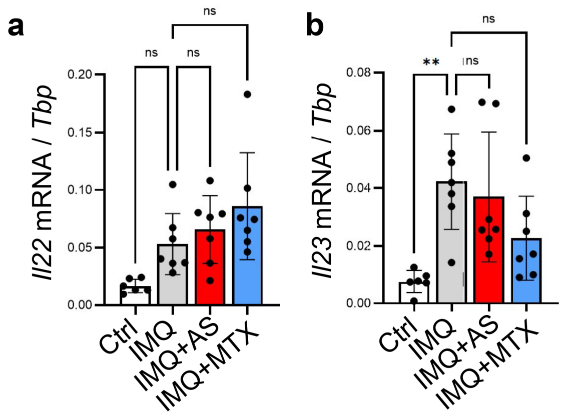

Figure S2. Administration of aluminum salt inhibited the development of Th1 and Th17 immune response in the IMQ-induced psoriasis model (Supplemental for main Figure 2): (a-b) qRT-PCR analysis of the mRNA expression levels of *Il22* or *Il23*(ratios to HK gene *Tbp* were shown) as indicated. All error bars indicate mean ± SEM (n=6~7/group) , and statistical analysis was performed by one-way ANOVA analysis with Dunnett-corrected multiple comparisons. **p < 0.01, ns, non-significant.

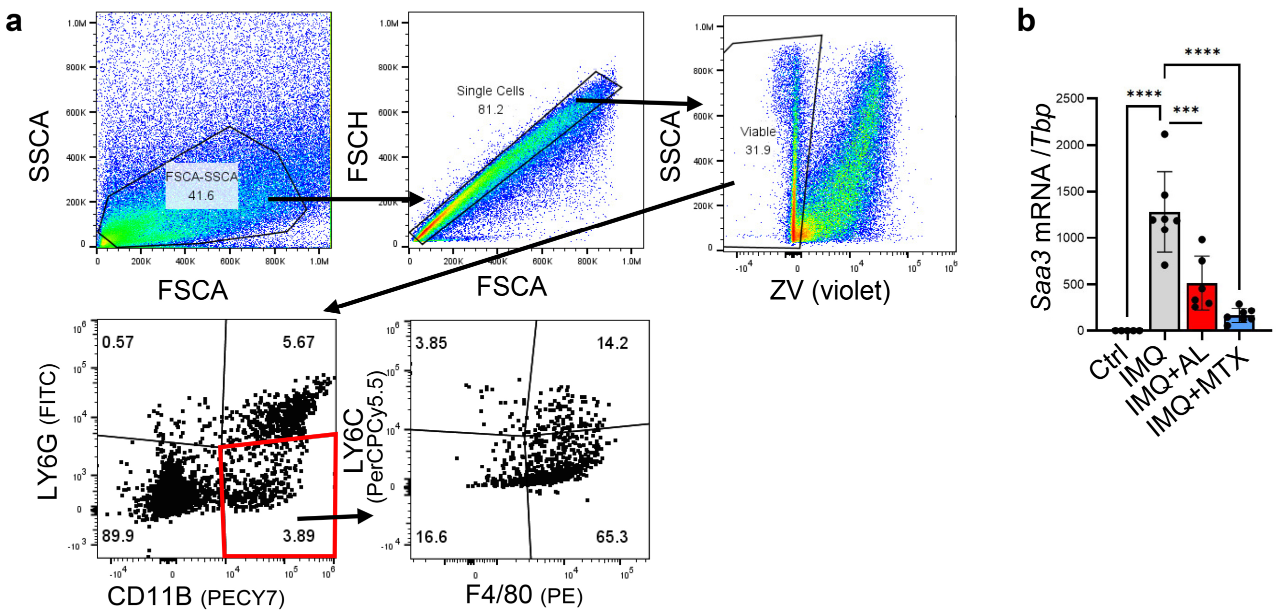

Figure S3. Administration of aluminum salt reduced the recruitment and activation of myeloid cells in the IMQ-induced psoriasis model (Supplemental for main Figure 3): (a) FACS gating strategy for analyzing the presence of neutrophils (CD11B+Ly6G+) and inflammatory macrophages (CD11B+Ly6G-F4/80+Ly6C+) in total skin cells; Description of what is contained in the first panel; (**b**) qRT-PCR analysis of the mRNA expression levels of *Saa3* (ratios to HK gene *Tbp* were shown) as indicated (n=6~7/group). All error bars indicate mean ± SEM (n=6~7/group), and statistical analysis was performed by one-way ANOVA analysis with Dunnett-corrected multiple comparisons. ***p < 0.001, ****p < 0.0001.

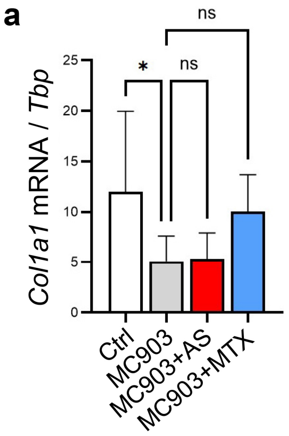

Figure S4. Administration of aluminum salt promoted the development of MC903-induced atopic dermatitis-like skin inflammation (Supplemental for main Figure 4): (a) qRT-PCR analysis of the mRNA expression levels of *Col1a1* (ratios to HK gene *Tbp* were shown) as indicated. All error bars indicate mean ± SEM (n=6~7/group) , and statistical analysis was performed by one-way ANOVA analysis with Dunnett-corrected multiple comparisons. **p < 0.01, ns, non-significant.

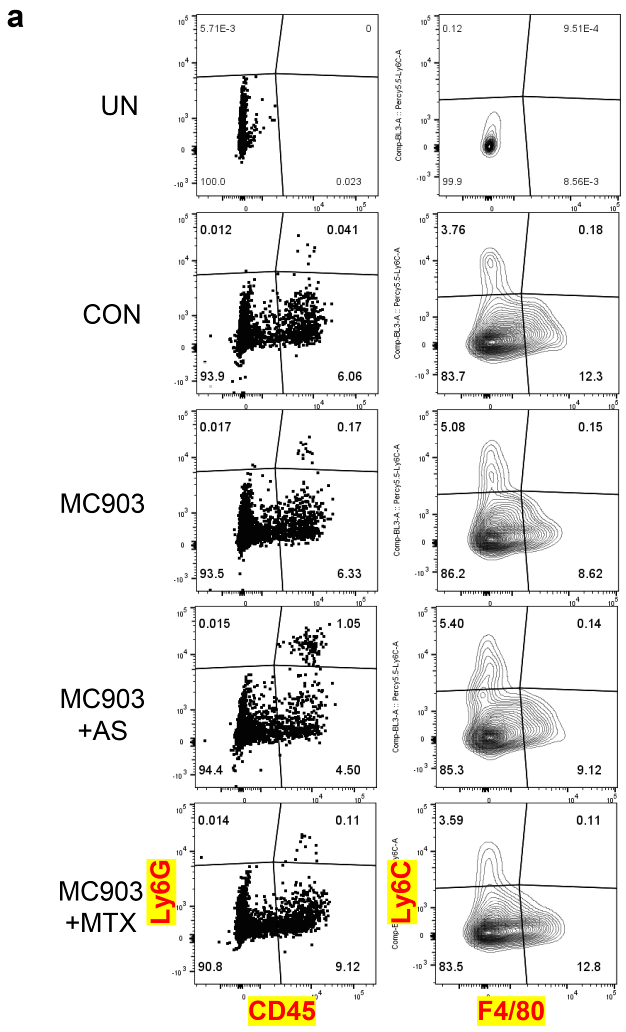

Figure S5. Effect of aluminum salt injection in modulating the MC903-mediated activation of T cells or myeloid cells (Supplemental for main Figure 5): (a) FACS plots analyzing the presence of neutrophils (CD11B+Ly6G+) and inflammatory macrophages (CD11B+Ly6G-F4/80+Ly6C+) in total skin cells

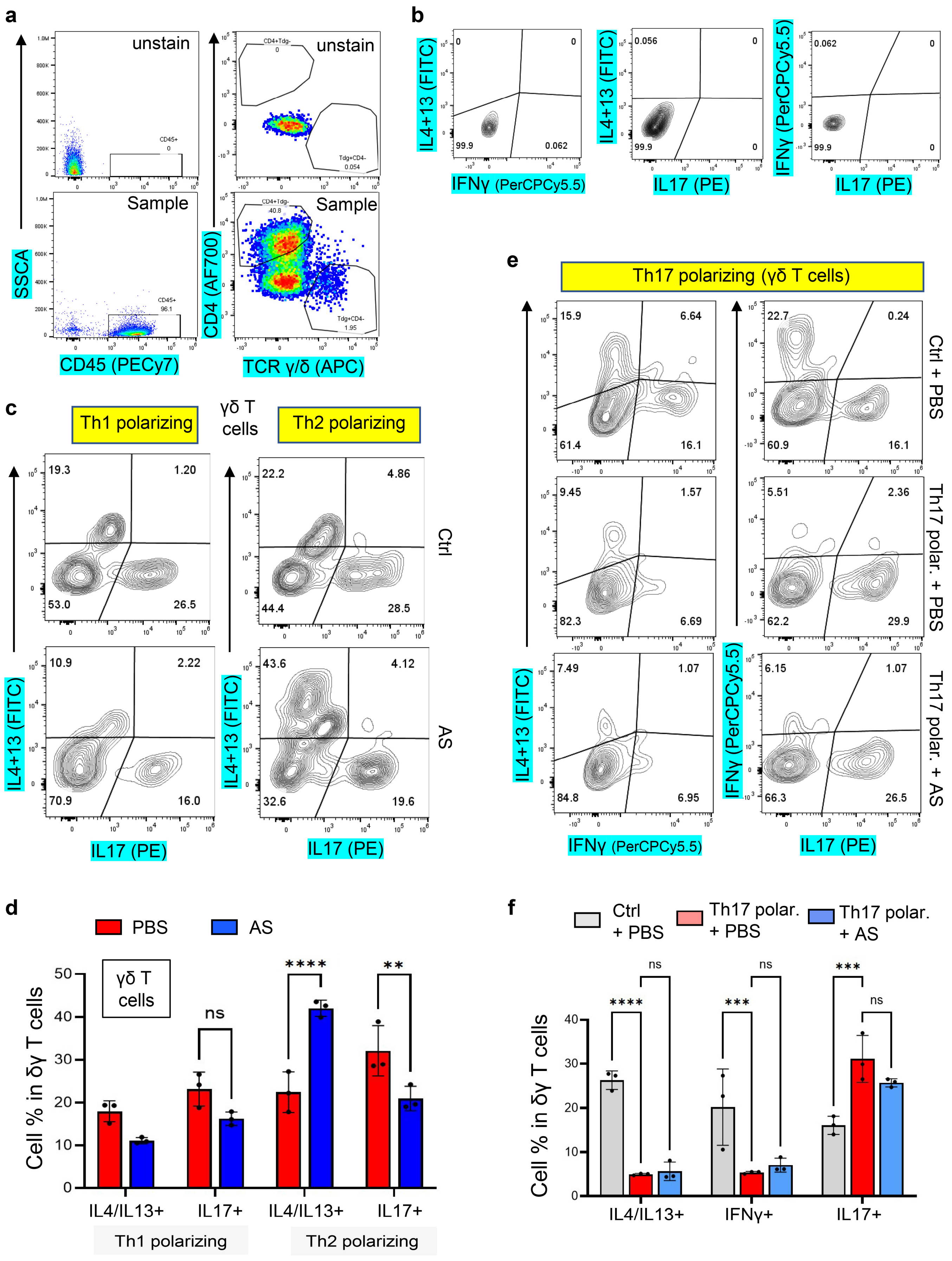
a
unstain
Sample
SSCA
CD45 (PECy7)
CD4 (AF700)
TCR γ/δ (APC)
b
IL4+13 (FITC)
IFNγ (PerCPCy5.5)
IL17 (PE)
c
Th1 polarizing
γδ T cells
Th2 polarizing
Ctrl
AS
IL4+13 (FITC)
IL17 (PE)
d
PBS
AS
γδ T cells
Cell % in δγ T cells
ns
****
**
IL4/IL13+
IL17+
Th1 polarizing
Th2 polarizing
e
Th17 polarizing (γδ T cells)
Ctrl + PBS
Th17 polar. + PBS
Th17 polar. + AS
IL4+13 (FITC)
IFNγ (PerCPCy5.5)
IL17 (PE)
f
Ctrl + PBS
Th17 polar. + PBS
Th17 polar. + AS
Cell % in δγ T cells
****
***
ns
IL4/IL13+
IFNγ+
IL17+

Figure S6. The in vitro effect of aluminum salt in modulating T cell differentiation and myeloid cell activation (Supplemental for main Figure 6): (a) FACS gating strategy for analyzing $CD45^+$ $CD4^+$ T cells and $CD45^+$ γδ T cells in the skin; (b) unstained controls for indicated FACS plots; (c) FACS plots analyzing the expression of IL4/IL13, IFNγ or IL17As in $CD45^+$ γδ T cells under Th1- or Th2-polarizing condition; (**d**) quantified results (FACS plots shown in Fig. S6C) showing the percentage of $IL4/IL13^+$, $IFN\gamma^+$ or $IL17A^+$ cells in $CD45^+$ γδ T cells under Th1- or Th2-polarizing condition; (**e**) FACS plots analyzing the expression of L4/IL13, IFNγ or IL17As in $CD45^+$ γδ T cells under Th17-polarizing condition; (**f**) quantified results (FACS plots shown in Fig. S6C) showing the percentage of $IL4/IL13^+$, $IFN\gamma^+$ or $IL17A^+$ cells in $CD45^+$ γδ T cells under Th17- polarizing condition;). All error bars indicate mean ± SEM (n=3/group) , and statistical analysis was performed by two-way ANOVA with Bonferroni-corrected multiple comparisons. *$p < 0.05$, **$p < 0.01$, ***$p < 0.001$, ****$p < 0.0001$, ns, non-significant.
